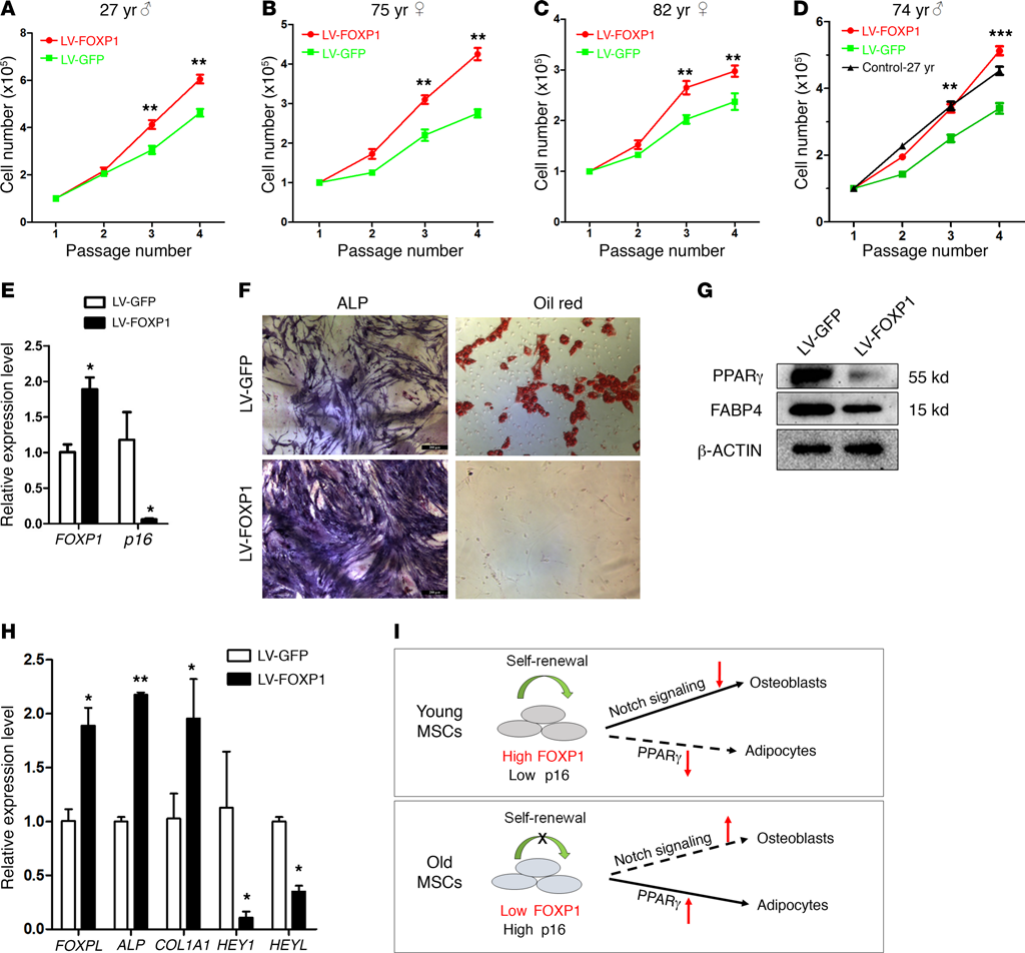# FOXP1 controls mesenchymal stem cell commitment and senescence during skeletal aging

**DOI:** 10.1172/JCI191424

**Published:** 2025-02-17

**Authors:** Hanjun Li, Pei Liu, Shuqin Xu, Yinghua Li, Joseph D. Dekker, Baojie Li, Ying Fan, Zhenlin Zhang, Yang Hong, Gong Yang, Tingting Tang, Yongxin Ren, Haley O. Tucker, Zhengju Yao, Xizhi Guo

Original citation: *J Clin Invest*. 2017;127(4):1241–1253. https://doi.org/10.1172/JCI89511

Citation for this corrigendum: *J Clin Invest*. 2025;135(4):e191424. https://doi.org/10.1172/JCI191424

In Figure 7G of the original article, there was an error in the β-actin blot, which was an inadvertent duplication of the β-actin blot in Figure 5G. The corrected figure 7, based on the original source data, is provided below. The supplemental material has been updated online with the correct unedited blot images. The HTML and PDF versions have been updated. 

The authors regret the error.

## Supplementary Material

Supplemental data

## Figures and Tables

**Figure 7:**