# Supplementary material for: FOXP1 controls mesenchymal stem cell commitment and senescence during skeletal aging
Source: J Clin Invest. 2025 Feb 17;135(4):e191424. doi: 10.1172/JCI191424 (PMC11827802; doi:10.1172/JCI191424)

Supplemental figures and Figure legends

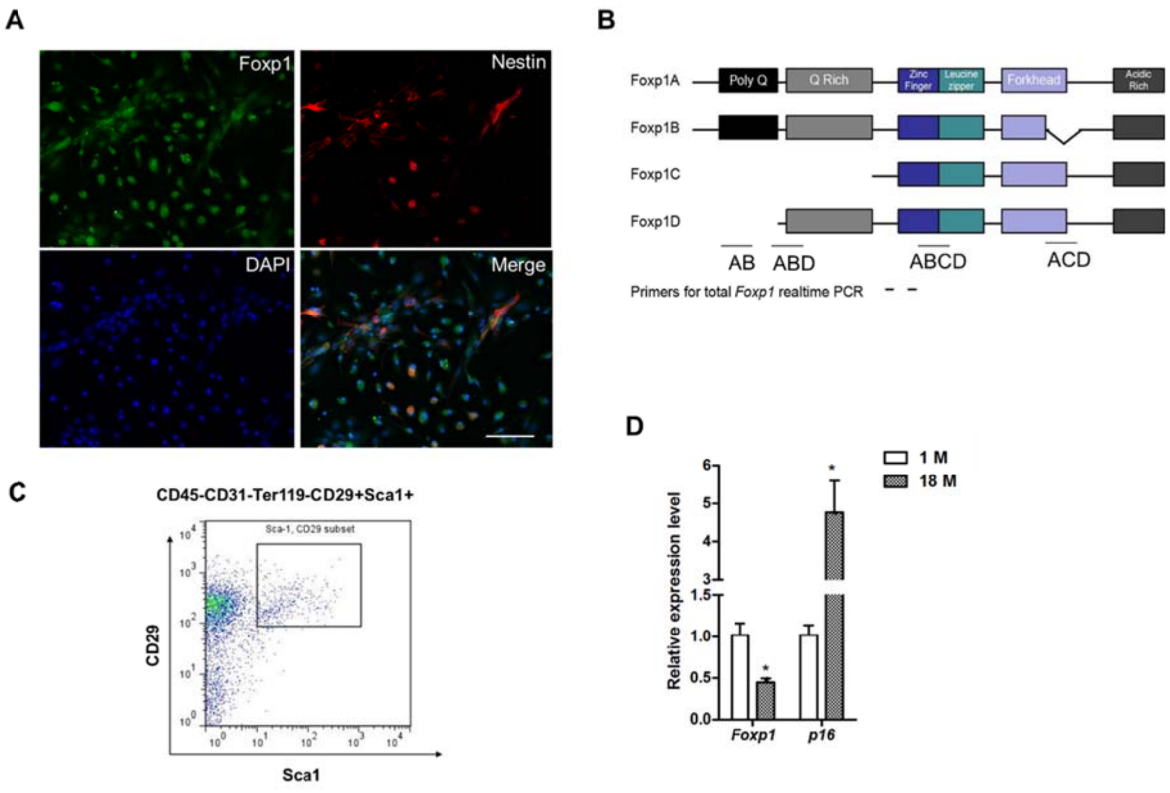

Supplemental Figure S1. Expression of Foxp1 during MSC differentiation.

(**A**) Co-localization of Foxp1 and Nestin in cultures of MSCs isolated from 1-month-old bone marrow (BM). (**B**) Locations of oligonucleotide probes used for qPCR detection of Foxp1A, B, C, and D isoforms. (**C**) Representative dot plot of MSC FACS employing expression (+) or absence (-) of standard markers: $CD45^-$ $CD31^-$ $Ter119^-$ $CD29^+ Sca1^+$. (**D**) qPCR detection of Foxp1 and p16 in MSCs sorted by FACS in BM from 1 and 18- months-old donors.

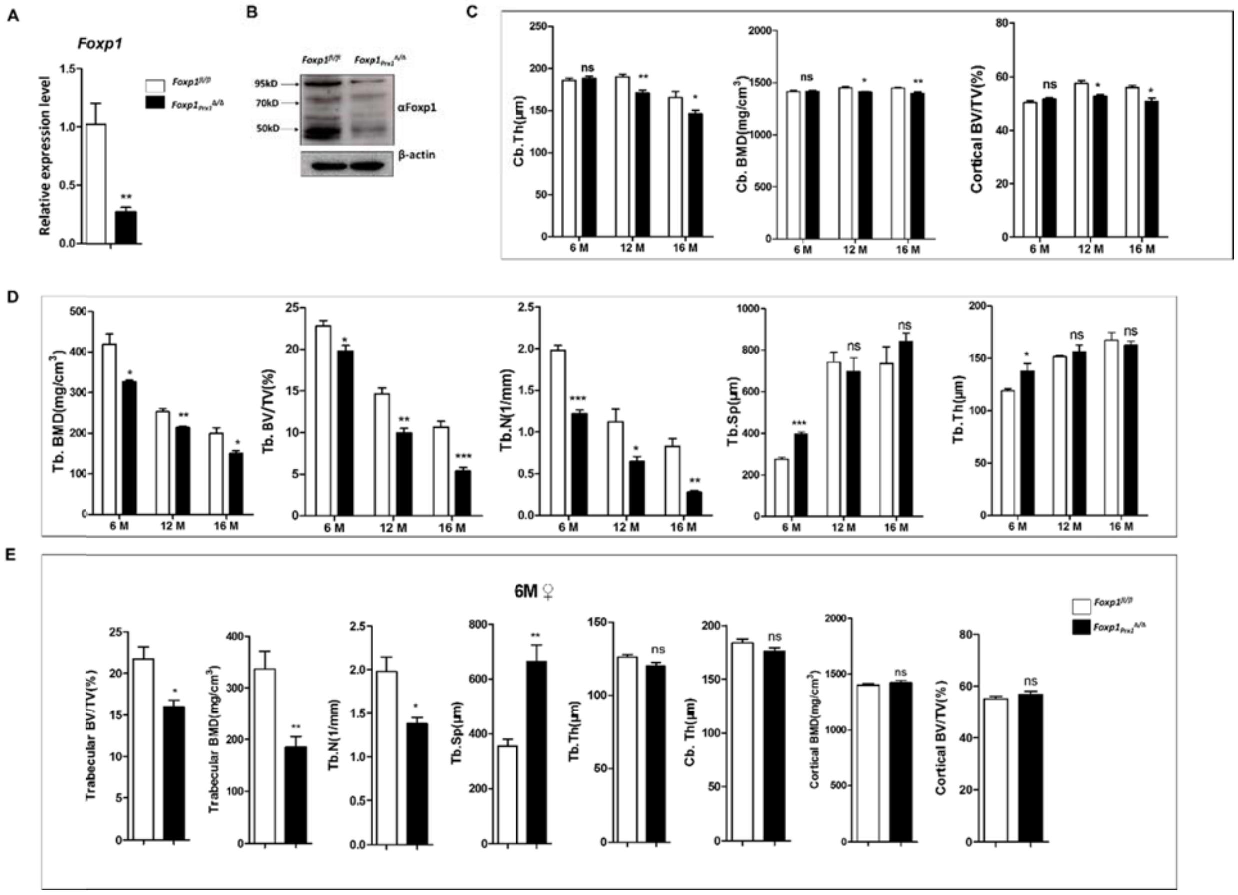

Supplemental Figure S2. Bone properties of *Foxp1*$_{Prx1}^{\Delta/\Delta}$ female mutants.

(**A, B**) Assessment of *Foxp1* expression in BM MSCs from *Foxp1*$_{Prx1}^{\Delta/\Delta}$ mutants by qPCR and western blotting. **, *P*<0.01; n=4. (**C, D**) μCT analyses detects progressive loss of volume, number, and BMD of trabecular bones as well as volume, thickness, and BMD of cortical bones in *Foxp1*$_{Prx1}^{\Delta/\Delta}$ mutants between 6 and 16 months. n=4. (**E**) The volume, number and BMD of trabecular bones were decreased, whereas trabecular separation was increased in mutants as compared to controls. The thickness of trabecular bones and parameters of cortical bones were unaltered. Abbreviations: BV/TV, bone volume/tissue volume; BMD, bone mineral density; Tb.N., trabecular bone number; Tb.Sp., trabecular bone separation; Tb.Th., trabecular bone thickness; Cb.Th., cortical bone thickness. *indicates *P*<0.05; **, *P*<0.01; ***, *P*<0.001; n=4.

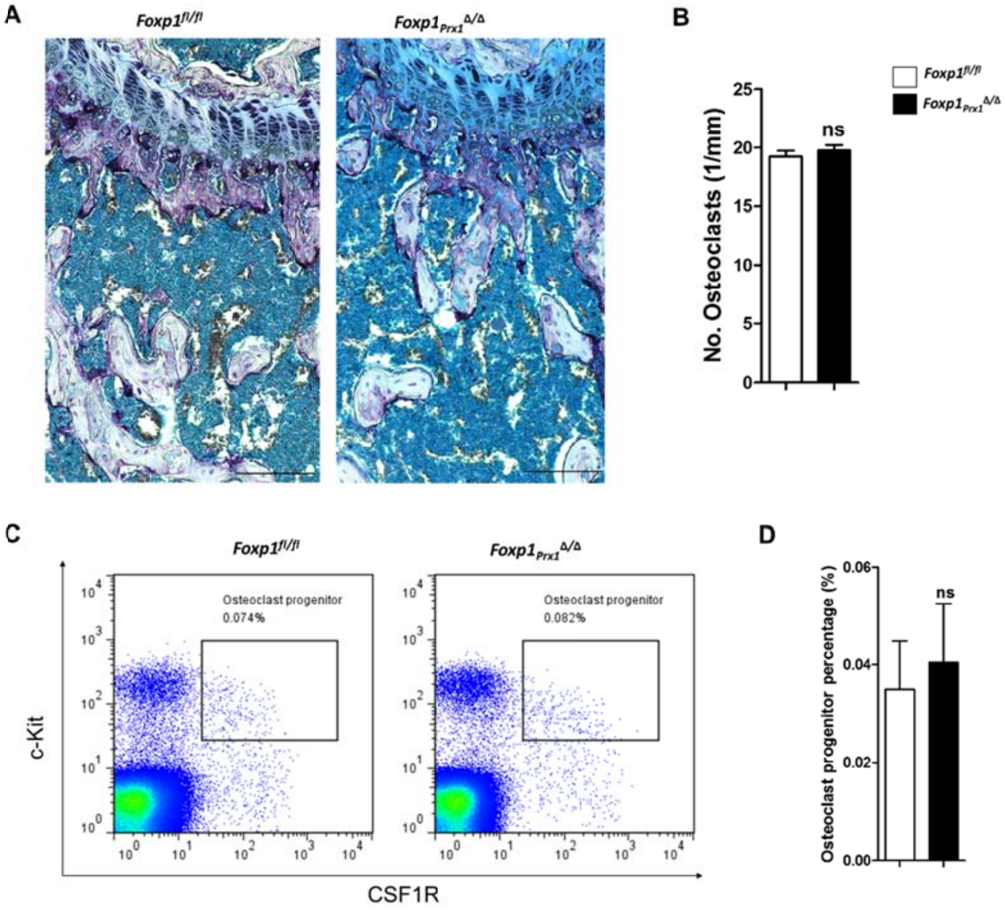

Supplemental Figure S3. TRAP staining for osteoclasts in *Foxp1*$_{Prx1}^{\Delta/\Delta}$ mice.

(**A**) TRAP staining for trabecular bones of Foxp1 mutant and control mice at 3 of age. (**B**) Quantification of osteoclast numbers in (**A**). (**C**) Dot plot of FACS analysis of osteoclast progenitor cells in BM *Foxp1* mutant and control mice at **3** months of age as defined by FACS expression (+) or absence (-) of standard markers: $CD3^-B220^-CD11b^-CSF1R^+cKit^+$. (**D**) Quantification of osteoclast progenitor cell numbers in (**C**); ns, nonsignificant; n=3.

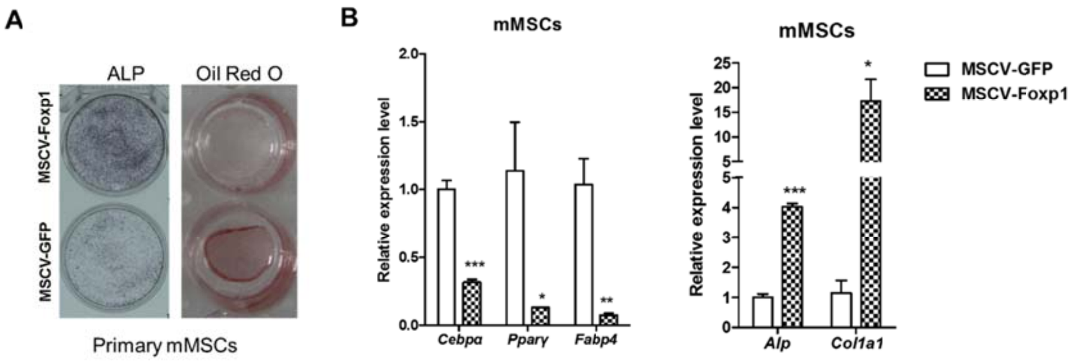

Supplemental Figure S4. Foxp1 regulates the differentiation of adipocytes and osteoblasts in MSCs.

(**A**) *Foxp1* was overexpressed in murine MSCs by insertion into retrovirus (pMSCV-Foxp1) followed by stable-transfection of MSCs and then selected and cultured in differentiation medium. Cell differentiation was assessed 6 days after adipogenic induction by oil red O staining or 14 days after osteogenic induction by ALP staining. (**B**) The expression of adipogenic markers (*CEBP*α, *PPAR*γ and *Fabp4*) analyzed by RT-qPCR 6 days following adipogenic induction of Foxp1-expressing MSCs cells. (**C**) Expression of bone markers (*Alp* and *Col1a1*) as assessed by RT-qPCR following 14 days of osteogenic culture. *, $P<0.05$; ***, $P<0.001$; n=3.

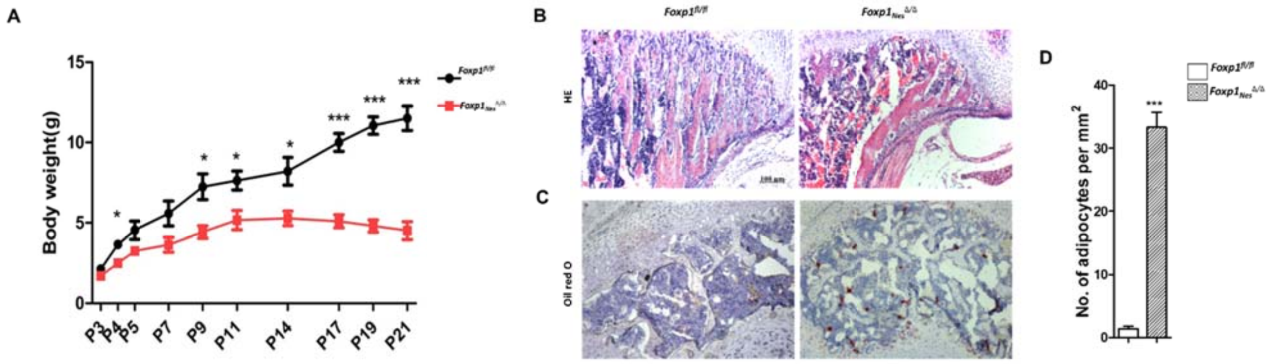

Supplemental Figure S5. Histological analysis of the bone marrow in *Foxp1*$_{Nes}^{\Delta/\Delta}$ mice.

(**A**) Growth curve of *Foxp1*$_{Nes}^{\Delta/\Delta}$ mice from measured at days after passage (P) from P3 to P21. (**B**) H&E staining of tibias from *Foxp1*$^{fl/fl}$ and *Foxp1*$_{Nes}^{\Delta/\Delta}$ mice at P21. Representative images were taken from the primary ossification region below the growth plates. (**C**) Oil red O staining of the tibias of (B) at P21. Representative images were taken from the secondary ossification proximal to the knee joint. (**D**) Quantification of the adipose droplets in (C); ***, $P<0.001$; n=5.

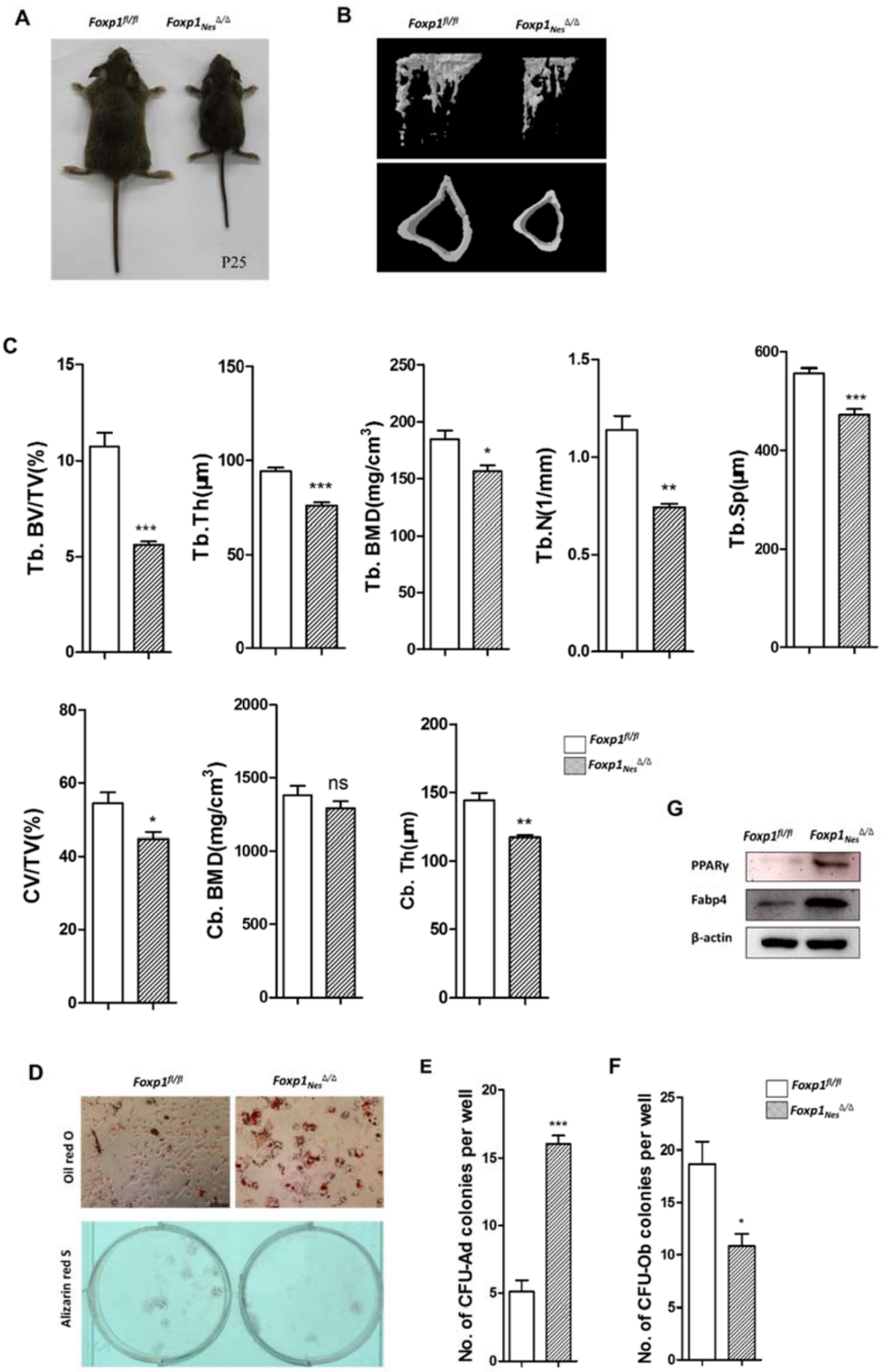

Supplemental Figure S6. The altered osteogenic and adipogenic potency of MSCs in *Foxp1*$_{Nes}^{\Delta/\Delta}$ mice.

(**A**) Representative images of *Foxp1*$_{Nes}^{\Delta/\Delta}$ and *Foxp1*$^{fl/fl}$ mice at P25. (**B**)

Representative images of μCT analysis for trabecular and cortical bones of tibias in *Foxp1*$_{Nes}^{\Delta/\Delta}$ at P21. (**C**) Quantification of bone properties indicating that trabecular BV/TV, BMD, Tb.N, Tb.Th and Tb.Sp were decreased, whereas BS/BV was relatively increased. Similarly, Cb.Th and CV/TV of the cortical bones were also decreased but cortical BMD was not significantly altered. *, P<0.05; **, P<0.01; ***, P<0.001; n=5. (**D**) The adipogenic potency of BM mesenchymal progenitors was assessed 14 days after adipogenic induction by oil red O staining (upper panel). Osteogenic differentiation of mesenchymal progenitors was assessed by Alizarin red staining at 21 days of differentiation induction (lower panel). (**E, F**) Quantification of adipocyte colonies (CFU-Ad) and osteoblast colonies (CFU-Ob) in (**D**). *, P<0.05; ***, P<0.001; n=4. (**G**) Western blot identification of PPARγ and Fabp4 expression in BM mesenchymal progenitors from *Foxp1*$_{Nes}^{\Delta/\Delta}$ and *Foxp1*$^{fl/fl}$ mice at P21.

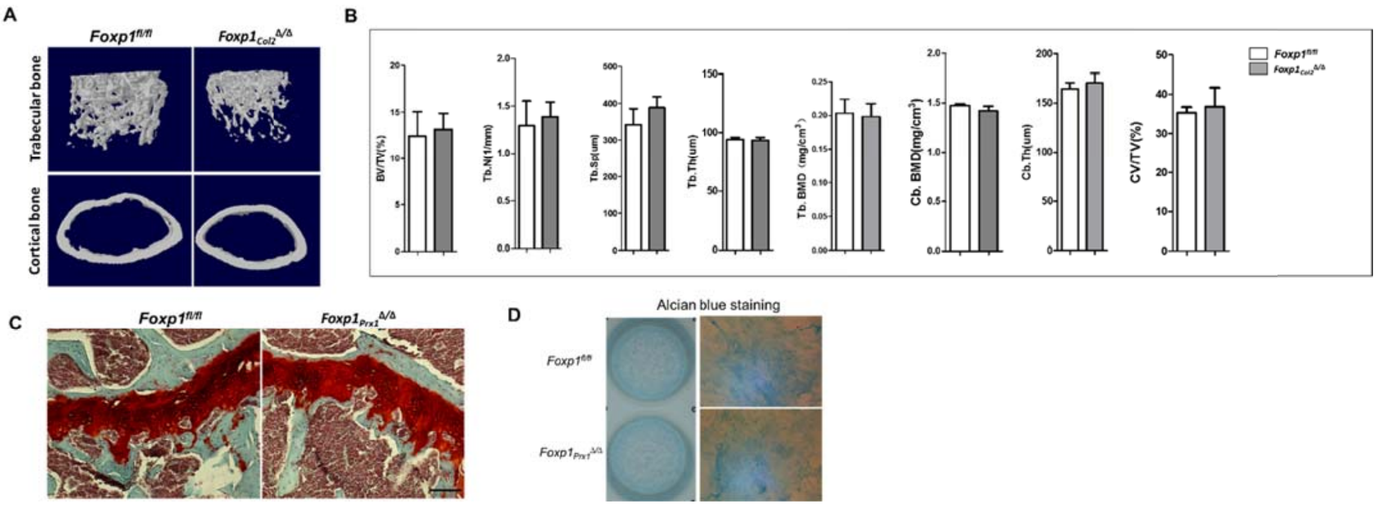

Supplemental Figure S7. Bone phenotypes in the *Foxp1*$_{Col2}^{\Delta/\Delta}$ mice.

(**A**) Representative images of trabecular and cortical bones from tibia of *Foxp1*$_{Col2}^{\Delta/\Delta}$ mutant and *Foxp1*$^{fl/fl}$ control 3 months old mice by μCT analyses. (**B**) μCT analyses detect no changes in bone properties in *Foxp1*$_{Col2}^{\Delta/\Delta}$ mutant mice. Abbreviations: BV/TV, bone volume/tissue volume; BMD, bone mineral density; Tb. N., trabecular bone number; Tb. Sp., trabecular bone separation; Tb. Th., trabecular bone thickness;

Cb. Th., cortical bone thickness. *, P<0.05; **, P<0.01; ***, P<0.001; n=5. (**C**) Safranin O staining of the growth plate of $Foxp1_{Prx1}^{\Delta/\Delta}$ mutant and $Foxp1^{fl/fl}$ controls at 8 months of age. (**D**) Alcian blue staining for the chondrogenic differentiation of mesenchymal progenitor cells after 14 days induction.

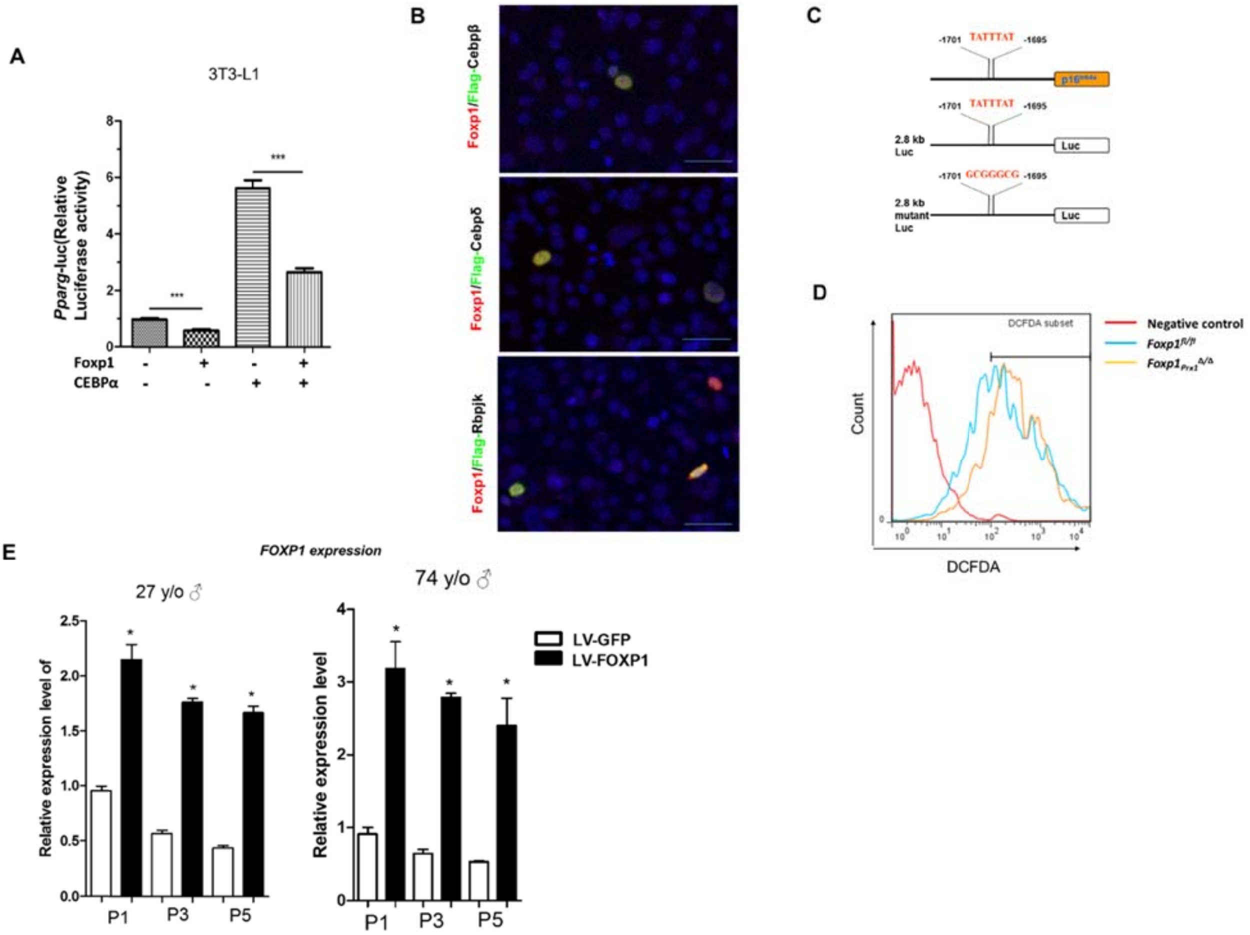

Supplemental Figure S8. Foxp1 represses the transactivation of *PPARγ*-Luc by CEBPα.

(**A**) Foxp1 repressed the transactivation of *PPARγ*-Luc by CEBPα. *, P<0.05; **, P<0.01; ***, P<0.001; n=3. (**B**) Co-localization of Foxp1 with CEBPβ, CEBPδ or RBPjκ in the nuclei of C3H10T1/2 cells transfected with Foxp1 and CEBPβ/δ-Flag or RBPjκ-Flag expression vectors. Green, anti-Flag; red, anti-Foxp1; blue, DAPI

staining to identify nuclei. Bar, 50 µm. (**C**) Schematic diagram showing that the mutation induced within the p16 promoter, which was employed in Figure 6C. (**D**) Representative histograms of DCFDA mean fluorescence intensities in mesenchymal progenitors of the second passages of cultures. (**E**) Expression of *FOXP1* in P1, P3 and P5 passages of 27- and 74-year-old hMPCs following Foxp1 lentiviral-mediated overexpression.

Oligos sequence for PCR

Primers for realtime PCR

Species	Name	Sequence
Mouse	β-actin-F	AGAGGGAAATCGTGCGTGACA
	β-actin-R	CACTGTGTTGGCATAGAGGTC
	Foxp1-F	TCTCGTCCTCGGCACCTT
	Foxp1-R	GTCACAAACCGCCTCACA
	Nestin-F	CACACCTCAAGATGTCCC
	Nestin-R	GAAAGCCAAGAGAAGCCT
	Cebpα-F	TGGACAAGAACAGCAACGAG
	Cebpα-R	TCACTGGTCAACTCCAGCAC
	PPARγ-F	GGAAAGACAACGGACAAATCAC
	PPARγ-R	TACGGATCGAAACTGGCAC
	Fabp4-F	GATGAAATCACCGCAGACGACA
	Fabp4-R	ATTGTGGTCGACTTTCCATCCC
	p21-F	GAACATCTCAGGGCCGAAAAC
	p21-R	CTGCGCTTGGAGTGATAGAA
	p27-F	ACTAACCCGGGACTTGGAGA
	P27-R	GAAATTCCACTTGCGCTGAC
	p53-F	GTCACAGCACATGACGGAGG
	p53-R	TCTTCCAGATACTCGGGATAC
	p16-F	CTAGAGAGGATCTTGAGAAGAGGGC
	p16-R	TAGTTGAGCAGAAGAGCTGCTACGT
	Bmi-F	CTACACGCTAATGGACATTGCCT
	Bmi-R	CCATCCCTCTGGTGACTCATCTT
	Hey1-F	CACTGCAGGAGGGAAAGGTTAT
	Hey1-R	CCCCAAACTCCGATAGTCCAT
	HeyL-F	GAAGCGCAGAGGGATCATAGA
	HeyL-R	CCAATCGTCGCAATTCAGAA
	Jagged1-F	CTTCAATCTCAAGGCCAGCC
	Jagged1-R	CAGGCGAAACTGAAAGGCAG

	Alp-F	GCCTGGATCTCATCAGTATTTGG
	Alp-R	GTTCAGTGCGGTTCCAGACAT
	Col1α1-F	CCGGAAGAATACGTATCACC
	Col1α1-R	ACCAGGAGGACCAGGAAGTC
	Runx2-F	CCGGGAATGATGAGAACTA
	Runx2-R	ACCGTCCACTGTCACTTT
	Osterix-F	CTCTCTGCTTGAGGAAGAAG
	Osterix-R	GTCCATTGGTGCTTGAGAAG
Human	ALP-F	AACATCAGGGACATTGACGTG
	ALP-R	GTATCTCGGTTTGAAGCTCTTCC
	COL1A1-F	GTGCGATGACGTGATCTGTGA
	COL1A1-R	CGGTGGTTTCTTGGTCGGT
	HEY1-F	ATCTGCTAAGCTAGAAAAAGCCG
	HEY1-R	GTGCGCGTCAAAGTAACCT
	HEYL-F	GGAAGAAACGCAGAGGGATCA
	HEYL-R	CAAGCGTCGCAATTCAGAAAG
	ACTIN-F	CCAGCACAATGAAGATCAAGAT
	ACTIN-R	AGAAAGGGTGTAACGCAACTAA
	FOXP1-F	GGGGCAGTATGGACAGTGGATGA
	FOXP1-R	TTGAGAGGTGTGCAGTAGGCGTG

Primers for ChIP-PCR	
Name	Sequence
Pparg-ChIP-F	CCCATTGAGCTATTGCTTC
Pparg-ChIP-R	TCAGTGACTTGTGGACTTT
p16-ChIP-F	TACACAGTTATGAGTTAGGGCAA
p16-ChIP-R	CTTCTTGAGGTCTGTAAGGAAAA

Figure 1B

Foxp1

β-actin

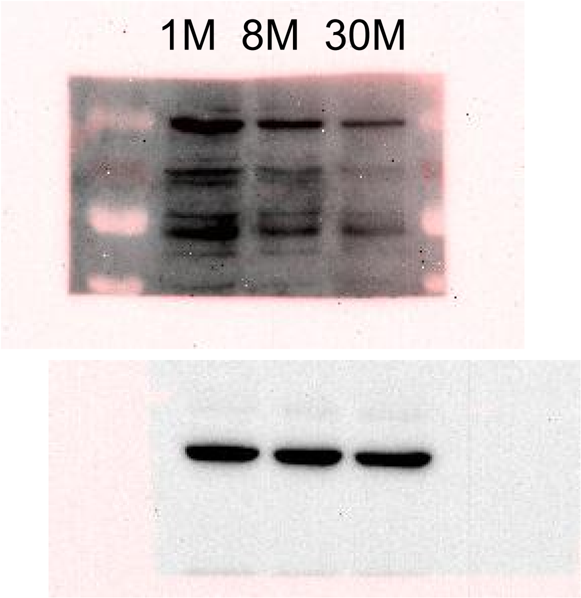

Figure 1E

Foxp1

β-actin

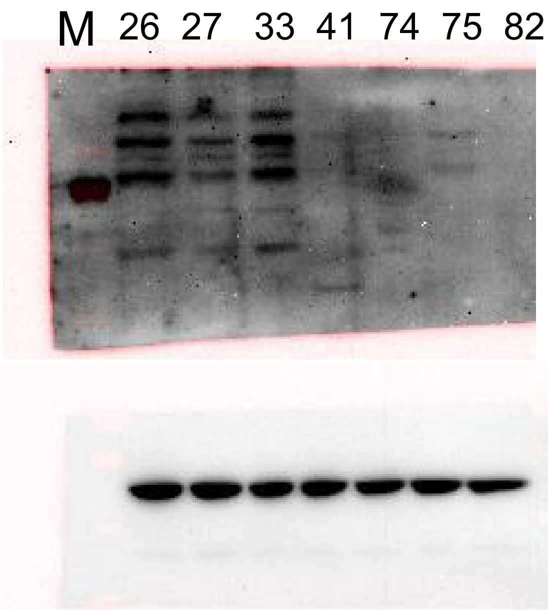

Figure 2B

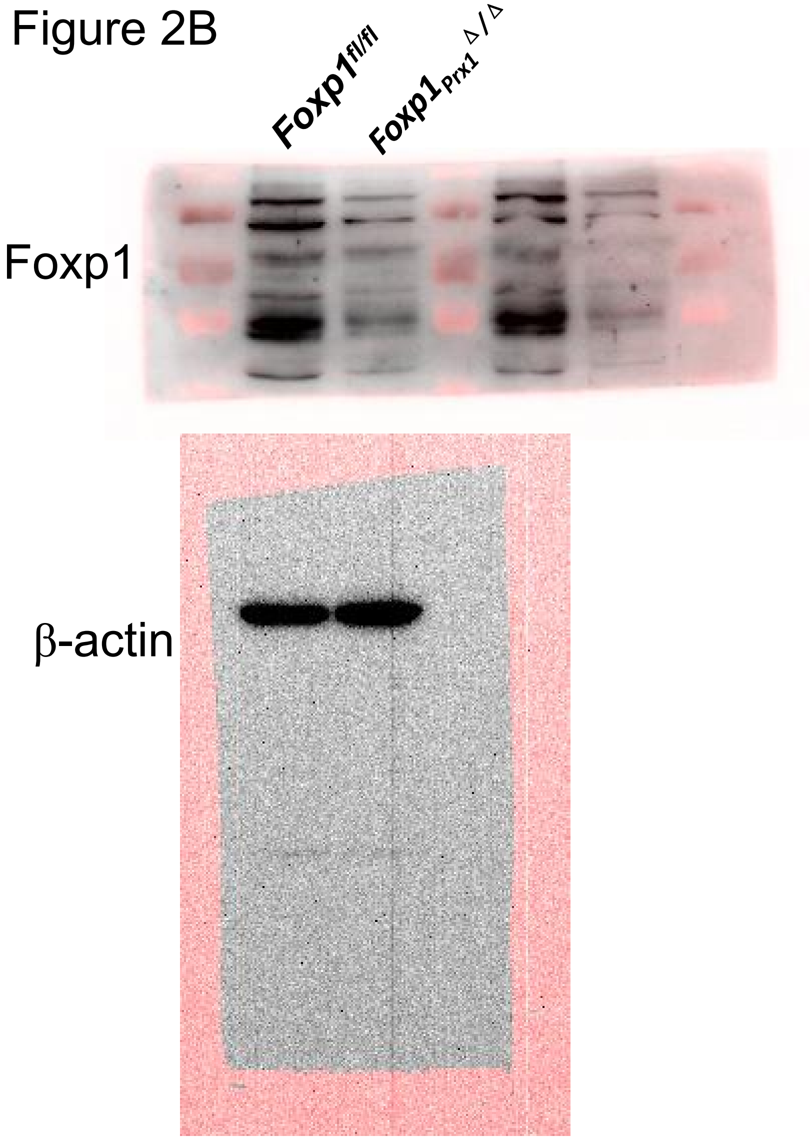

Figure 4F

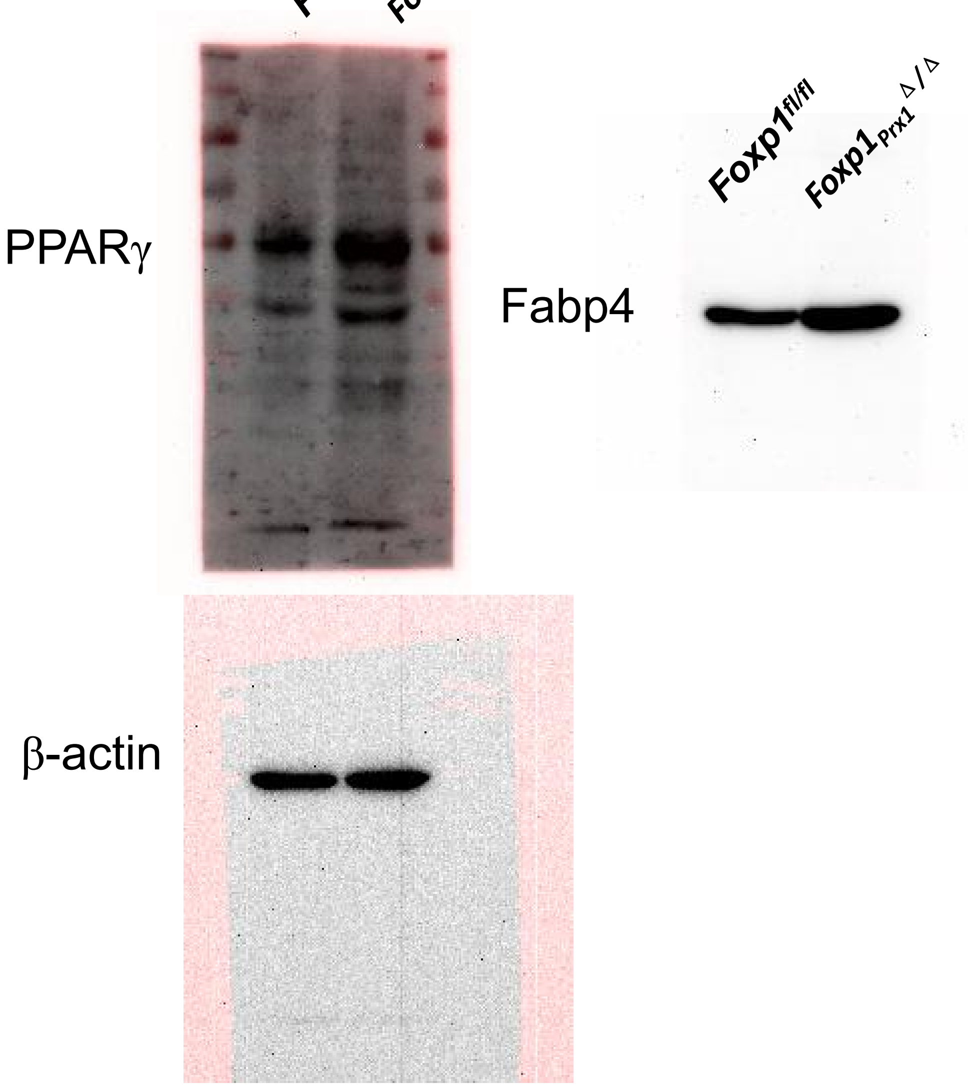

Figure 4A

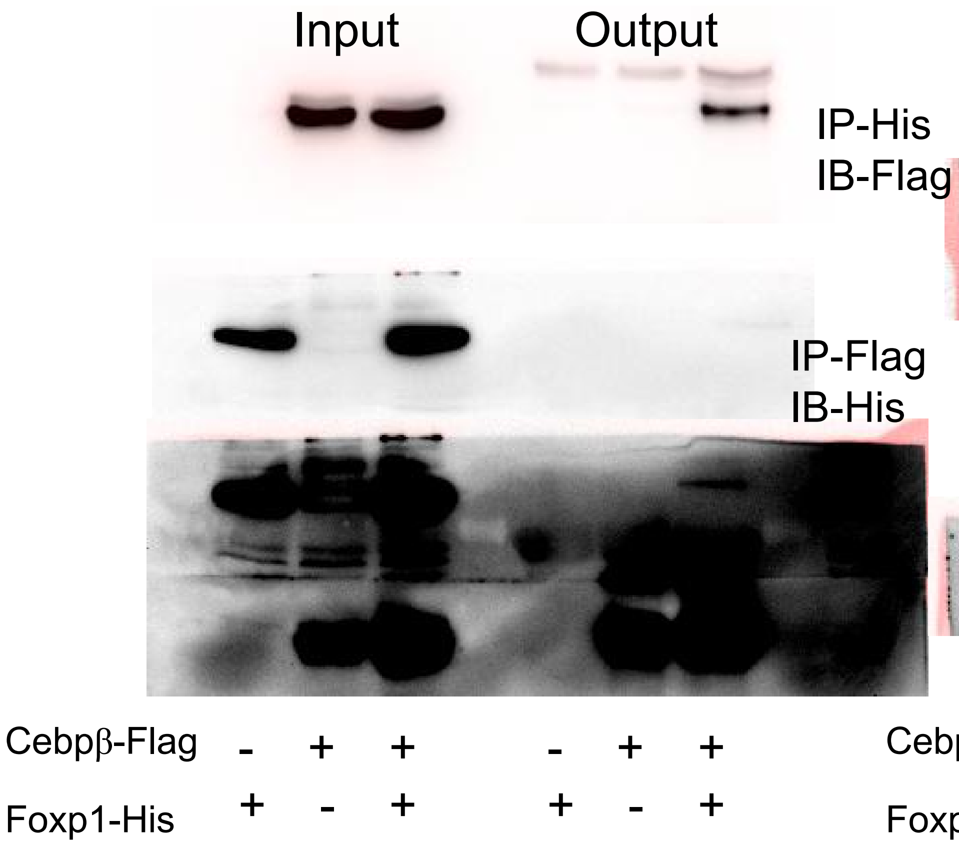

Figure 4B

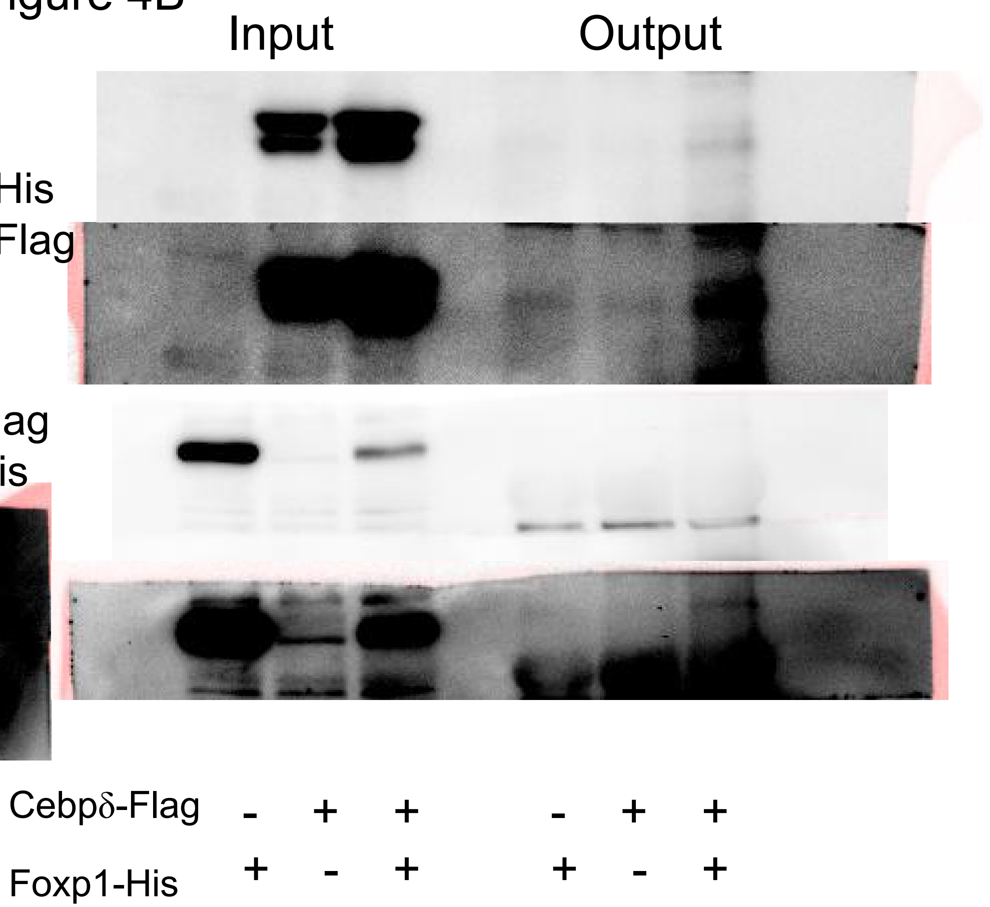

Figure 4C

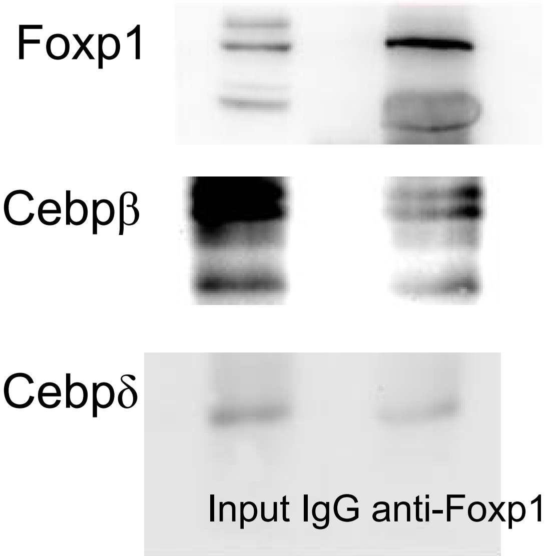

Figure 4K

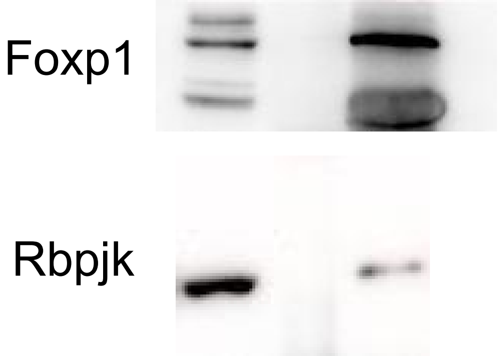

Figure 4J

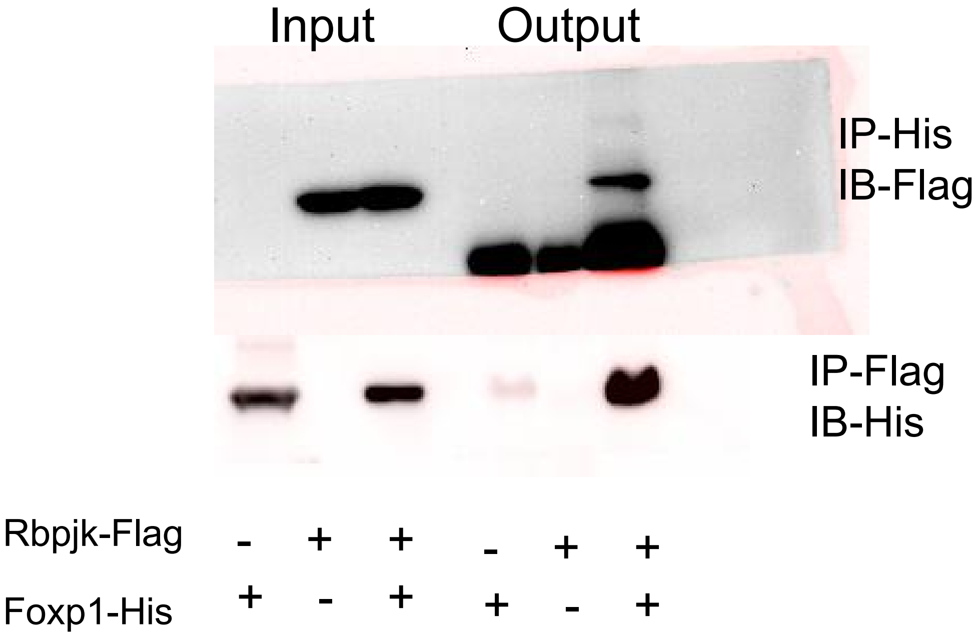

Figure 5G

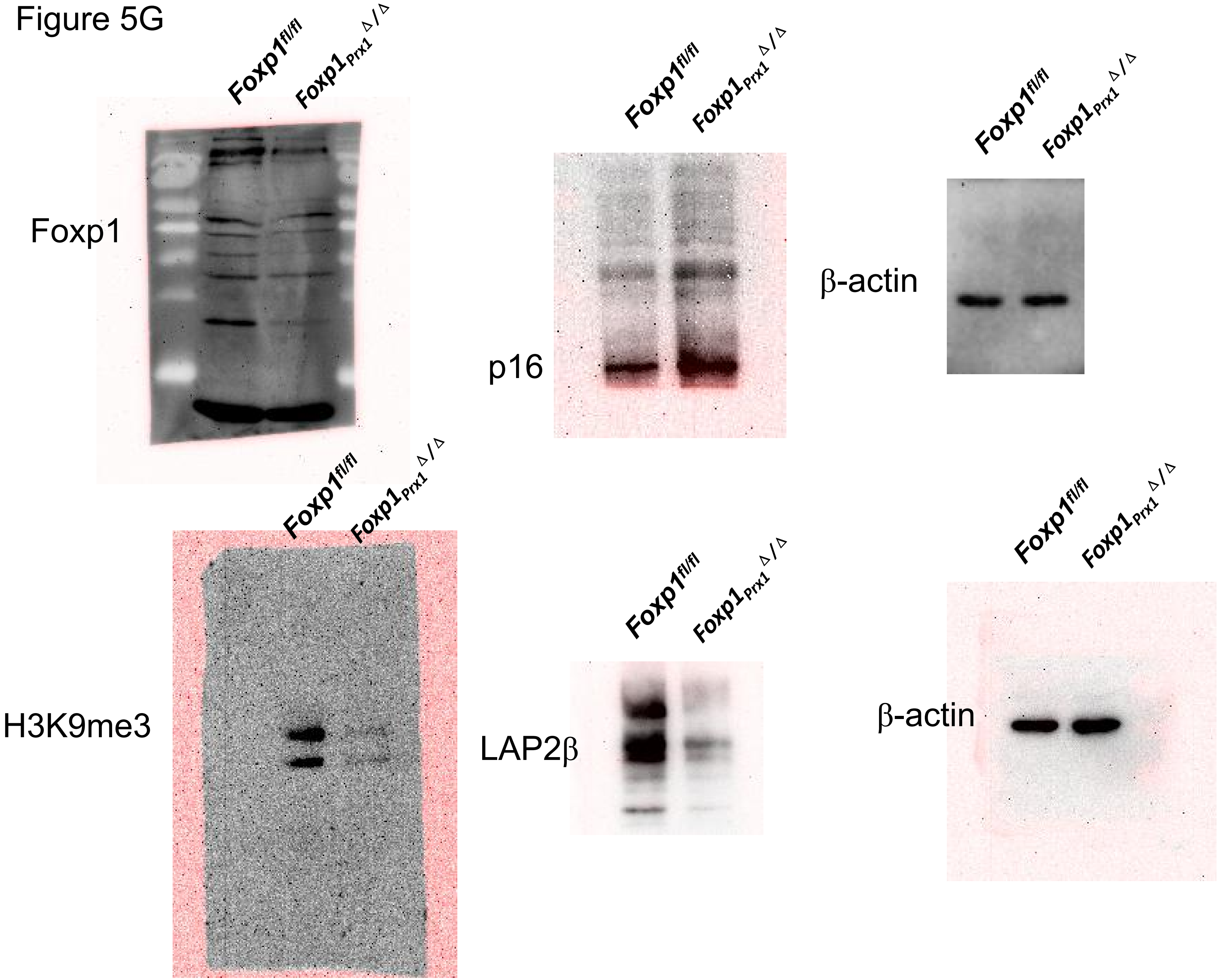
Foxp1fl/fl
Foxp1Prx1Δ/Δ
Foxp1
p16
β-actin
H3K9me3
LAP2β
β-actin

Figure 7G

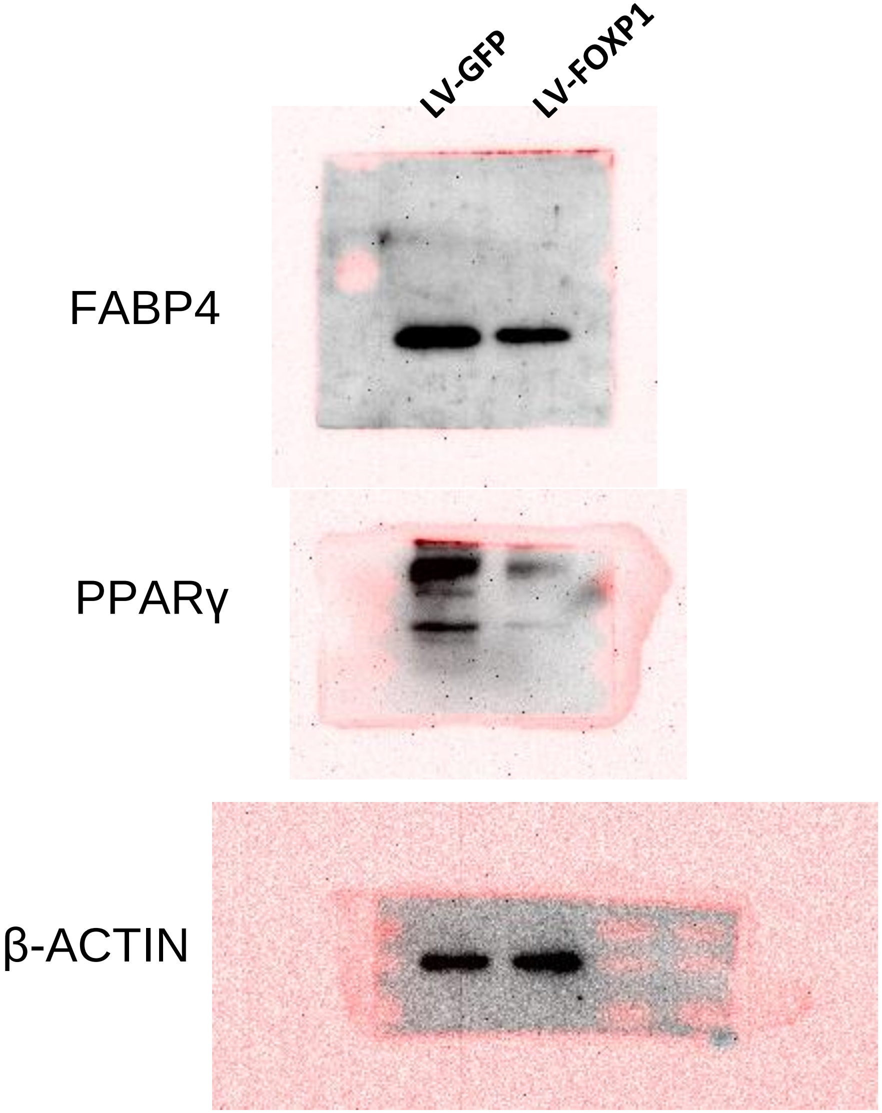

Figure S6G

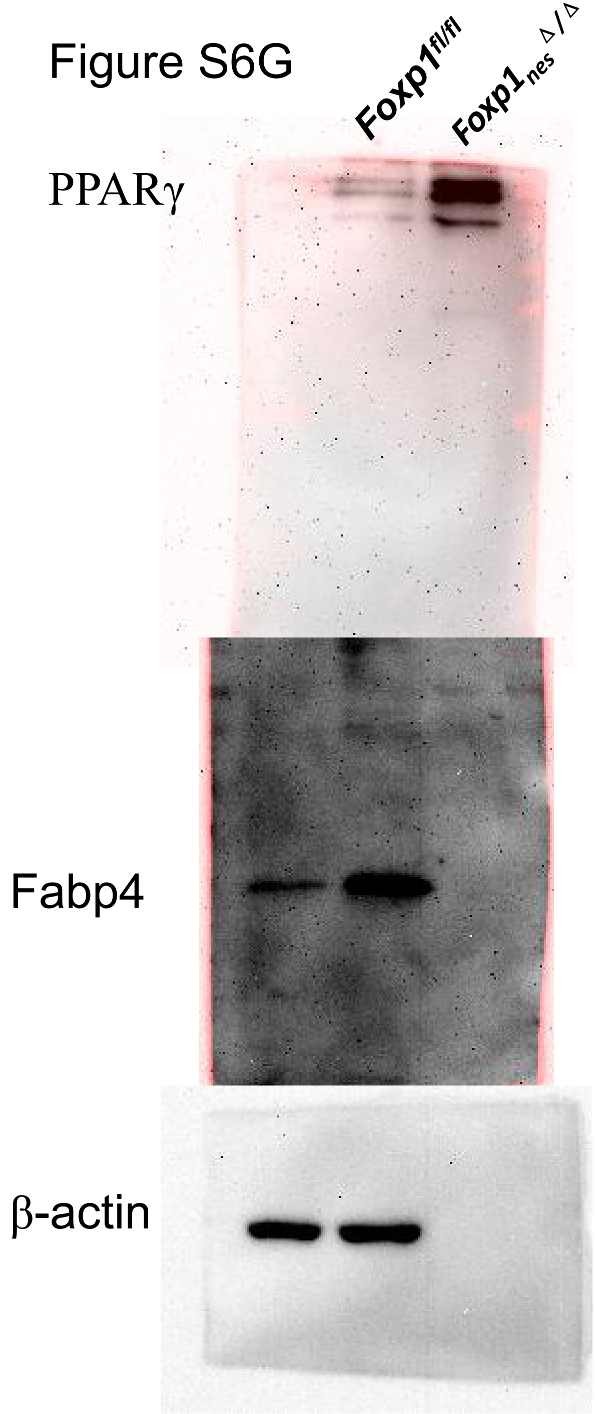

Supplement: Supplemental data [file jci-135-191424-s102.pdf]
